# The Provident Principle in Connection with Hospitals

**Published:** 1890-04-05

**Authors:** Edmund Hay Currie


### The Provident Principle in Connection with Hospitals.

By Sir Edmund Hay Currie.

It was with much pleasure that I read a short paper before the Hospitals Association on the eminently important matter of the application of the Provident Principle to Hospital administration, convinced, as I am, and as I have been for many years, of the immensely beneficial results which would accrue upon the adoption of an intelligent system based upon this principle, at every hospital in London.

It is my firm conviction that if the authorities of any one of the great London hospitals would apply such a system, merely as an experiment, for a sufficient length of time to fully test its merits, its complete adoption in all the London hospitals would only become a matter of time and of the mastery of such minor obstacles as change of custom and, in some cases, of amendment of charter.

A complete reconstruction of hospital methods would be the natural corollary of the verdict of public opinion when once the possibility was demonstrated on a large and obvious scale, of helping the deserving sick poor without either pauperisation to the patients or injury to the medical profession.

As I have implied, there are difficulties, and possibly in some cases serious difficulties, in the way, but before what I conceive to be the urgent present necessity of amending the out-patient arrangements of our great hospitals, these difficulties, formidable in themselves as they may be, become of minor importance, and should certainly never be allowed to constitute obstacles to, at any rate, the trial of what would be undoubtedly a very great measure of reform.

It is a matter of the widest notoriety, and one which only the wilfully blind can refuse to see, that the benefits of the London hospitals are greatly abused, and that, more especially in the out-patient department, large numbers of persons obtain medical relief who can well afford to pay the fees of medical practitioners in the usual way, and who can in no respect be considered fit recipients of public charity.

The manifold harm worked by the attendance of these patients is obvious. The accommodation of hospitals is becoming more severely taxed every day, and the places of the really poor are being usurped by those who have no right to them.

Each of these patients shuts out some poor and deserving person, diverts from its proper course some portion of the noble charity funds of this city, and injures the medical profession by retaining in his pocket the fees which should in justice have been paid to a doctor. This class of abuse has become so widespread, and has existed so long, that I have no doubt that hundreds of the more well-to-do class of patients accept things as they stand, and altogether fail to realise the serious wrongs which they, taking advantage of a defective system, inflict upon the cause of charity.

It has become among them a matter of essential, every-day faith that an illness of any kind means a visit to the hospital, and the notion of paying a doctor never enters their heads, and these, too, are the people who would probably, at the first blush, indignantly repudiate the imputation of being recipients of charity; the hospital is to them a general medical convenience, and never presents itself to their minds as a charity.

I believe that the evil wrought by this class of patients is the result of the want of thought to a large extent, and that they never realise its gravity. It may perhaps be said that persons of this description, though perfectly able to pay for ordinary medical advice, may wish at some time to consult some eminent specialist whose regular fees are beyond their means, but whom they may see at the hospital without payment, and although it may be said that in the matter of medical advice, as in other matters, poor men may be expected to stand at a disadvantage relatively to rich men, there is, nevertheless, nothing in the scheme which I purpose bringing under your notice which would prevent special arrangements being made for cases of this description.

The scheme which I have in my mind, and which seems to me, at present, to be the most suitable in its general features to effect the necessary results, is not a visionary, theoretical, and untried one. It has been fully and carefully tested at one of our smaller hospitals (the Metropolitan Hospital in the Kingsland Road) with the most complete success, and it is, in my opinion, only because it has been tried at a small hospital, and not at one of the great central institutions, such as that, a room of whose premises we are now occupying, that the system or something very similar to it is not now in course of becoming general among us. I will endeavour to describe the practice at the institution I have named.

To begin with, a certain district was mapped out, over which it was proposed that the operations of the hospital should extend; the hospital was a small one, placed in a district previously unprovided for, and it was therefore necessary to confine its work to local needs. All that part of Kingsland and the surrounding districts of Shoreditch, Haggerston, Hoxton and Bethnal Green, comprised within a circle with a radius of one mile, with a population of 260,000 persons, with the hospital as centre, was fixed upon as the area to be provided for.

The former out-patient department was formed into a "Provident" Department under the following conditions, which, I may parenthetically remark, it has not been found necessary to alter in principle in any particular. We determined broadly in the first place that only those who were really too poor to pay the ordinary fees of a medical man should be allowed to join the Provident Department, and that such persons should be made to pay *during health and sickness alike*, some nominal weekly or monthly sum by way of subscription. The exact course adopted was this: No applying out-patient was ever sent away. He was seen by the doctor or surgeon on duty, prescribed for, and given sufficient medicine to last a week; but before he could be attended to again it was necessary for him, after showing his eligibility, to join the Provident Department.

To be eligible he must, first of all, reside within the district; if a single man, he must not be in receipt of an income of more than 21s. per week, and if married and supporting his family, of more than 35s. per week. Women had to satisfy the same conditions. The fees charged were—for adults, one penny a week, or fourpence a month each; for children (who were not allowed to join without one, at least, of their parents), twopence a month each. Parents were, however, given the opportunity of compounding for all the children under the age of sixteen in one family for a subscription of sixpence a month.

An entrance fee of sixpence was charged for a single person, or for a family all joining at the same time, but this fee was remitted to members of benefit societies upon production of their cards of membership. The subscriptions, it should be most particularly noted, were payable during health and sickness alike, with a view of establishing habits of providence and forethought among the patients.

There is made an additional charge of one penny, as a sort of registration fee, each time a prescription is made up, quite irrespective of the quantity of medicine, all medicines being, of course, provided by the hospital.

The numbers of poor persons who have joined the Provident Department during the two years and two months the system has been in operation, are now dealt with during sickness in the manner following. The district is divided into four sections, each under the care of a doctor attached to the Provident Department. These four gentlemen are local medical practitioners, who receive a fixed salary from the hospital, in return for which each attends there one morning and one evening in each week, and makes home visits to patients when necessary. In ordinary cases the patient attends at the hospital in the usual way on the morning or evening when his doctor is present. If, however, he be too unwell to leave home, he may send for the doctor of his district, who, provided the message arrives before ten o'clock in the morning, visits the patient during the day; it is found necessary to make some additional charge in cases where the request for a home visit is not received until after ten o'clock. Should the doctor consider the condition of the patient too serious for treatment at home he recommends his admission as an in-patient.

The doctors of the provident department pass on to the honorary staff of the hospital all such cases as in their opinion require greater individual care and attention than can be given to ordinary out-patients.

The subscriptions of the provident members last year amounted to £653 2s. 10d., the salaries £425 12s. 6d., leaving a margin (roughly speaking) of £230 towards the cost of drugs, &c.

Married women are attended during their confinements by certificated midwives for a fee of 15s., which may be paid in instalments, and the midwife may, wherever necessary, call in the doctor without additional charge.

Such, briefly, is the system in use at the Metropolitan Hospital, and it is a system the success of which the governors of that institution have every reason to congratulate themselves upon. It excludes those whose means should place them above charity, and sends them to their proper resource, the regular medical practitioner.

It inculcates a certain self-respect among the very poor who are benefited by it by exacting a small payment for the attention they receive, and fosters habits of prudence and thrift among them by teaching them to provide, while in health, against the sickness which is sure to come some day. That these moral results are actually attained, and are not a mere supposition, is made very evident by the increasing regularity and steadiness of the subscriptions paid, as well as by their increasing numbers.

Of course attempts are made to abuse the system, just as is the case with every charitable system, and it is just possible that these attempts, in one or two cases, may have been successful, but such cases, if they have occurred at all, must have been few indeed. Of course the investigations made into the merits in each case before admitting a member to the Provident Department extend beyond the mere inquiries as to income and residence which I have mentioned in enumerating the leading features of the Metropolitan Hospital method, and the case would be an exceptional one indeed, in which any abuse of the benefits offered was at all an easy matter.

The amount realised by these small fees is, of course, not a very considerable one, but it much more than pays for the small additional clerical labour necessitated, the amount of such additional clerical labour depending, of course, very largely upon the degree of business ability brought to bear upon the regular collection of the subscriptions.

A very singular and, to me, unaccountable circumstance in connection with the establishment of this system at the Metropolitan Hospital, was the opposition which it encountered at the outset from some (certainly very few) medical men. We hear no more of this opposition now, however, the fact having by this time, seemingly, become visible to everybody that, as one would imagine was apparent upon the face of it, one of the first effects of the system was a beneficial one for the local medical practitioners.

In our poorer districts, as a rule, a medical man can hardly get a living within some distance of a hospital with a large out-patient department, and the exclusion from the benefits of the hospital of such of the out-patients as can afford to pay a doctor's fees, obviously means an extension of the practice o the medical men in the vicinity. But, in addition to this, there is another and a very important way in which this system benefits the profession. Every medical man is aware of the pernicious effects wrought in poor neighbourhoods by the establishment of the "doctor's shop" class. The poor using these places are just of the class who, under the provident system, go to our hospital and are thence passed on to the resident qualified practitioners appointed by the governors, who receive their salaries for attendance upon poor patients, who would in the ordinary course, not being able to pay their fees, either go as out-patients to a free hospital, or failing that, deliver themselves over to a "doctor's shop" to be poisoned at sixpence a visit.

All this would relieve the struggling local practitioner of the necessity of attempting to compete with the unscrupulous "doctor's shop" (the occupation of which would be gone), would raise his status, increase his practice, and, it seems to me, benefit him and the dignity of his profession in every way; and I am glad to be confirmed in my opinion by many distinguished members of the profession itself, who are much better qualified to give an opinion on this aspect of the subject than I am.

If this, or some similar plan were adopted generally, as I sincerely hope and trust it will be by the whole of the London Hospitals, it would be necessary to map out the metropolis into a system of districts, assigning each district its hospital or centre, and establishing a harmonious co-operation and intercommunication between all.

No part of London would then remain unprovided for, a complete system would be established wherewith to abolish the present great waste through abuses of the out-patient departments and the overlapping of the work of separate hospitals, and a habit of thrift would be encouraged among the very poor throughout the capital which might be trusted to go far toward remedying the improvidence and want of independent self-respect still, I fear, too prevalent among them.

